# Case Report: A rare case of mixed medullary-papillary thyroid carcinoma with mixed lymph node metastasis

**DOI:** 10.3389/fonc.2025.1676422

**Published:** 2025-11-13

**Authors:** De-shi Yang, Jing He

**Affiliations:** Department of Pathology, The Third Hospital of Mianyang, Sichuan Mental Health Center, Mianyang, Sichuan, China

**Keywords:** mixed medullary papillary thyroid cancer, medullary thyroid carcinoma, papillary thyroid carcinoma, pathological diagnosis, case report

## Abstract

**Introduction:**

By reviewing the diagnosis and treatment process of a rare case of mixed medullary-papillary thyroid carcinoma (MMPTC) in the right lobe, medullary thyroid carcinoma (MTC) combined with papillary thyroid carcinoma (PTC) in the left lobe, and multiple MTCs in the isthmus with mixed lymph node metastasis, we systematically analyzed its pathological features and immunophenotype.

**Case presentation:**

A 65-year-old Han male patient was found to have a hypoechoic nodule(TI-RADS category 5) in the right lobe of the thyroid gland during a physical examination. Additionally, enlarged lymph nodes were found in the right neck, accompanied by structural abnormalities (The morphology is regular, with a distinct boundary. The internal echo is heterogeneous, presenting anechoic areas and punctate hyperechoic foci. The lymph hilar structure is poorly visualized. Color Doppler Flow Imaging (CDFI) reveals punctate blood flow signals within and surrounding the lesion.). Serum calcitonin (CT), carcinoembryonic antigen (CEA), and thyroglobulin (TG) were all elevated. Subsequently, fine-needle aspiration cytology (FNAC) was performed on the right lobe thyroid mass and a biopsy was taken from the right cervical lymph nodes. The FNAC results indicated that the right lobe thyroid mass was suggestive of MTC. Meanwhile, metastatic PTC was found in the right cervical lymph node biopsy tissue.The patient subsequently underwent a total thyroidectomy combined with radical lymph node dissection in the thyroid and maxillofacial surgery department. The postoperative radical specimen showed that cancer foci were visible in the left lobe, isthmus and right lobe of the thyroid. Among them, a focus of MMPTC was found in the right lobe of the thyroid gland, with the tumor mainly composed of MTC components; in the left lobe, a coexisting lesion of PTC and MTC was found, the two foci are separated by normal thyroid tissue. Two foci of MTC were found in the isthmus of the thyroid gland. A total of 88 lymph nodes were dissected, among which 21 were found to have cancer metastasis. Postoperative serum CEA dropped to the normal level, and CT showed a significant decrease compared with that before the operation.

**Conclusions:**

The peculiarity of this case lies in the fact that preoperative serological tests showed abnormally elevated levels of CT, CEA and TG. FNAC of the right lobe of the thyroid indicated MTC, but at the same time, the puncture of cervical lymph nodes revealed the presence of PTC components. In such a situation, the possibility of coexistence of MTC and PTC should be highly suspected. A standardized surgical treatment plan should be selected, and during the pathological examination, standardized sampling and meticulous reading of the slides should be emphasized, combined with immunohistochemical detection, to reduce the risk of missed diagnosis, provide accurate pathological diagnosis, and thereby offer reliable basis for the formulation of postoperative treatment strategies and prognosis assessment.

## Introduction

According to statistics, thyroid cancer is the fastest-growing tumor in terms of cancer incidence in China, and the disease burden remains relatively heavy (1, 2). The 2020 Global Cancer Statistics Report shows that thyroid cancer ranks among the top ten most common cancers worldwide (3). Papillary thyroid carcinoma (PTC) accounts for more than 85% of all thyroid cancer cases and originates from follicular epithelial cells (4). Medullary thyroid carcinoma (MTC) originates from C cells or parafollicular cells and currently accounts for 4% to 8% of all thyroid cancers (5). The occurrence of these two types of tumors simultaneously is relatively rare, accounting for less than 1% of all thyroid tumors (6). Mixed subtype thyroid cancer (MSTC) refers to a malignant tumor in which two or more different types of cells are mixed within the same lesion of the thyroid. The incidence of MSTC is extremely low, accounting for approximately 0.13% of all thyroid cancers (7). Mixed medullary thyroid tumors (MMTs), including mixed medullary follicular thyroid carcinoma (MMFTC) and mixed medullary papillary thyroid carcinoma (MMPTC). MMPTC is a rare condition characterized by the concurrent presence of both MTC and PTC morphological and immunohistochemical features within the same lesion, where MTC and PTC are intricately interwoven. In contrast, synchronous PTC with MTC (PTC/MTC) refers to a scenario where the two components are separated by normal thyroid tissue (8). PTC and MTC exhibit distinct cellular origins, pathological characteristics, therapeutic approaches, and prognostic outcomes. Consequently, the concurrent occurrence of PTC and MTC, along with its accurate diagnosis, holds significant clinical importance.

In accordance with the guidelines established by the American Thyroid Association (ATA), patients presenting with concurrent MTC and PTC should undergo radical surgical intervention. This recommendation is predicated on the more aggressive nature of MTC compared to PTC, as well as the necessity for a more extensive surgical approach in MTC cases.The standard surgical procedure encompasses total thyroidectomy combined with bilateral central compartment lymph node dissection. The decision to perform ipsilateral and bilateral cervical lateral lymph node dissection is contingent upon imaging findings and serum calcitonin levels. Routine preoperative assessment of serum calcitonin (CT), carcinoembryonic antigen (CEA), and thyroglobulin (TG) levels facilitates the diagnosis of MTC lesions, while ultrasonographic characteristics aid in the identification of PTC lesions. The primary objective of preoperative examination is to ascertain the status of lymph node metastasis, thereby providing a basis for the selection of surgical approach and the determination of the extent of lymph node dissection. The therapeutic principle should be individualized based on the relative significance of the two tumor types. Given that patients with concurrent MTC and PTC exhibit characteristics of both malignancies, postoperative management may include radioactive iodine therapy, endocrine therapy, and targeted drug therapy (9). Several studies indicate that a subset of advanced-stage patients may achieve localized lesion remission through radiotherapy and chemotherapy; however, this observation necessitates validation through large-scale data analysis. The prognosis of patients is contingent upon a multitude of factors. Postoperative monitoring of serum CT, CEA, and TG, in conjunction with ultrasonographic examinations, is essential to enhance the detection rate of recurrent lesions. Patients with PTC exhibit a significantly higher 10-year relative survival rate, reaching 98%. Conversely, the prognosis of MTC is generally less favorable compared to PTC, with a 10-year survival rate of 80% (10). The prognosis of MMPTC falls between that of PTC and MTC, being inferior to PTC but superior to MTC.

The uniqueness of this MMPTC case lies in its concurrent presence of contralateral thyroid PTC/MTC, multiple MTCs in the isthmus, and ipsilateral lymph node metastasis with mixed carcinoma. To enhance the understanding of this condition among clinicians and pathologists, this article presents the case report accompanied by a literature review, aiming to provide valuable insights for pathological diagnosis and therapeutic management of the disease.

## Case presentation

**Figure 1:**
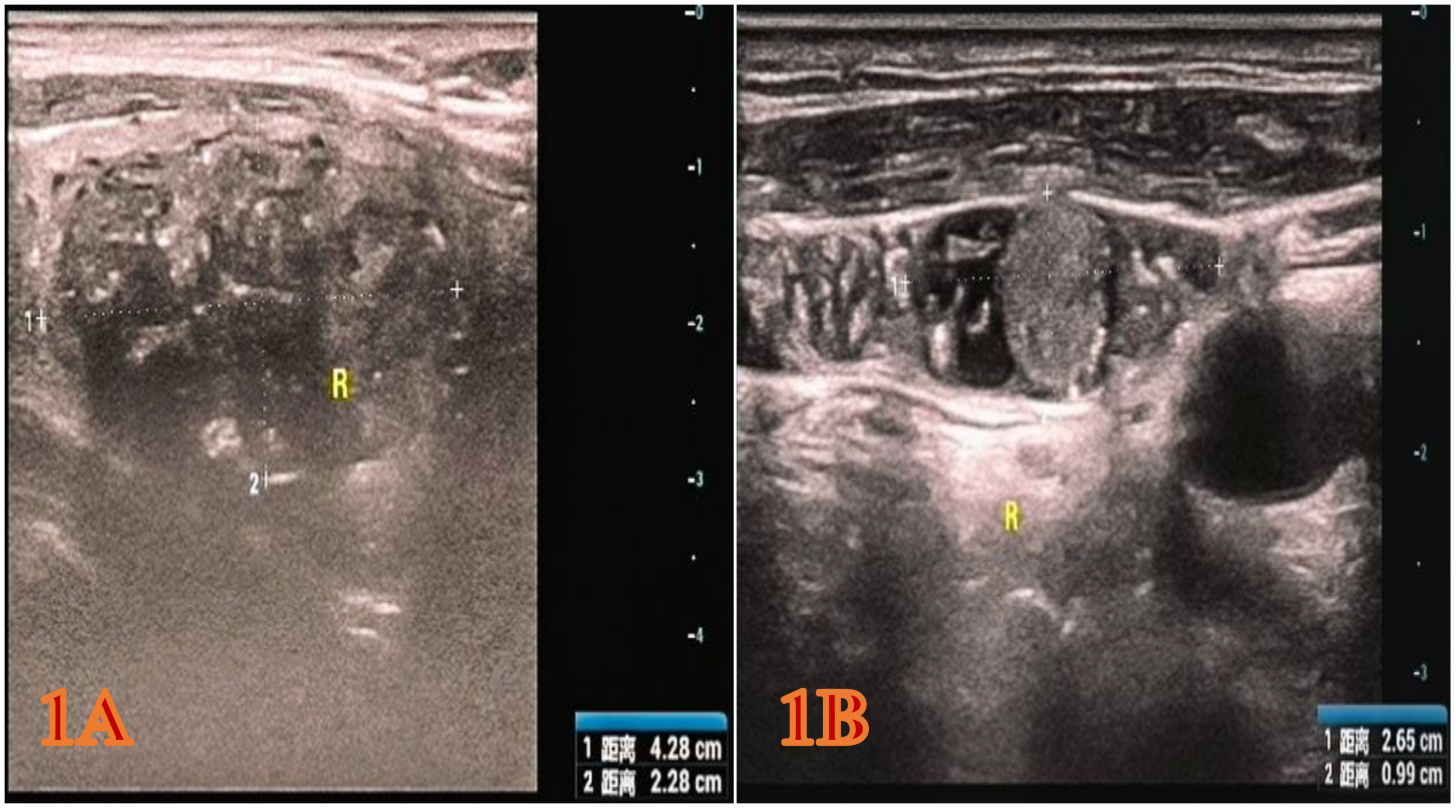
The color Doppler ultrasound image of the patient during the physical examination. **(A)** Right thyroid gland (hypoechoic nodule, 4.28x2.28x2.54 cm, heterogeneous hypoechoic, TI-RADS category 5). **(B)** Right cervical lymph nodes (the larger one approximately 2.65 x 0.99 cm, considered enlarged lymph nodes with structural abnormalities).

The patient is a 65-year-old Han male. During a physical examination at Mianyang Third People’s Hospital, a color Doppler ultrasound of the thyroid gland was performed, which revealed a hypoechoic nodule in the right lobe of the thyroid gland, measuring 4.28x2.28x2.54 cm (**Figure 1A**). The shape was relatively regular, with some unclear boundaries. Within it, scattered punctate and mass-like hyperechoic areas of varying sizes were observed. The TI-RADS classification was 5. A cystic nodule was also found in the left lobe of the thyroid gland, classified as TI-RADS 2. Color Doppler ultrasound of bilateral cervical and supraclavicular lymph nodes suggests an enlarged lymph node on the right side of the neck (the largest one measures 2.65x0.99 cm) with structural abnormalities (**Figure 1B**). The patient was subsequently admitted to the thyroid and maxillofacial surgery department for treatment. The patient has no history of tumors or family history of tumors in the past. Physical examination revealed a hard mass about 4.0 cm in diameter in the right lobe of the thyroid gland, with moderate mobility and no obvious tenderness. No obvious nodules were palpable in the left lobe of the thyroid gland, and no enlarged lymph nodes were palpable in the bilateral neck. Laboratory test results show that serum CT level is 1796.38 pg/ml, CEA is 81.69 ng/ml, and TG is 163.65 ng/ml. All these indicators are above the normal range. The results of thyroid function tests are normal.

Subsequently, fine-needle aspiration cytology (FNAC) of the right lobe thyroid mass and biopsy of the right cervical lymph nodes were performed under ultrasound guidance. In the ultra-thin liquid-based cytology (LCT) examination of the right thyroid lobe mass, although the HE staining under the microscope suggested MTC, the immunohistochemistry showed that CT was only focally weakly positive. Therefore, the final pathological diagnosis tended to be MTC, and it was recommended to further confirm through the postoperative radical specimen. However, the biopsy of the right cervical lymph node only revealed metastasis of PTC. The above results suggest that the patient may have two types of tumors, MTC and PTC, simultaneously. Therefore, a total thyroidectomy combined with radical lymph node dissection was performed.

**Figure 2:**
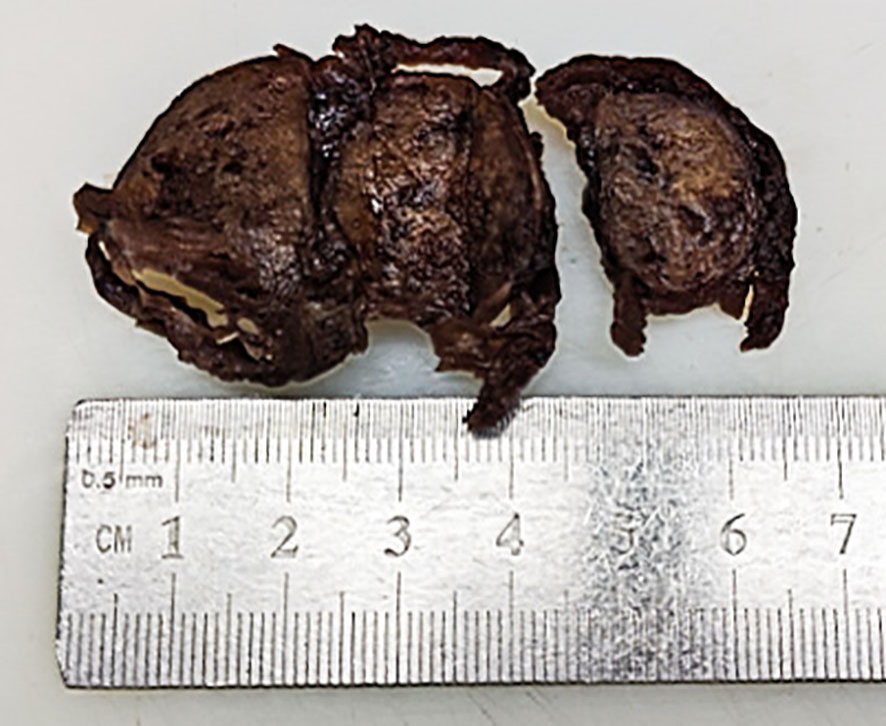
Gross image of the right lobe thyroid mass in the postoperative radical specimen.

**Figure 3:**
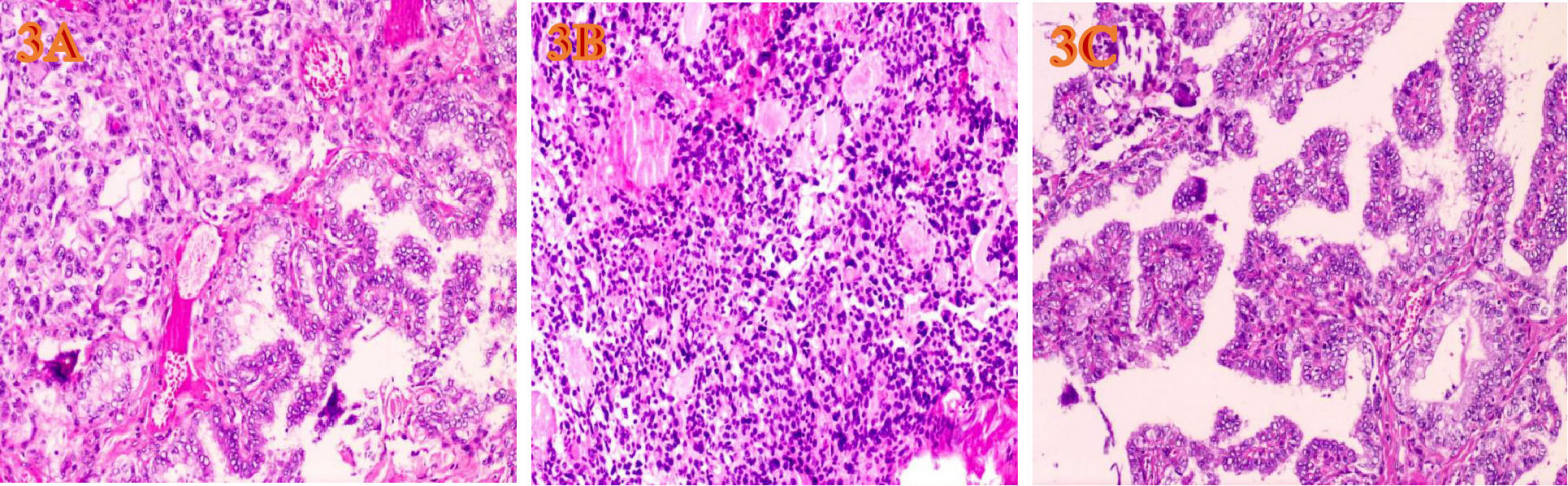
HE-stained microscopic images of tumors(H&E, ×200, magnification. **(A)** Under the microscope of MMPTC HE, MTC and PTC are interwoven. **(B)** The PTC cells are closely arranged, the cytoplasm is ground-glass-like, the nuclei are round or oval, and nuclear overlap, nuclear grooves and intranuclear pseudoinclusions can be seen. **(C)** The MTC cells have diverse shapes, including round, oval, and polygonal. Abnormal large cells and binucleated or multinucleated cells are observed. The nuclei are of varying sizes, with irregular nuclear membranes. The nuclear chromatin is granular (salt and pepper-like), and the nucleoli are prominent.

**Figure 4:**
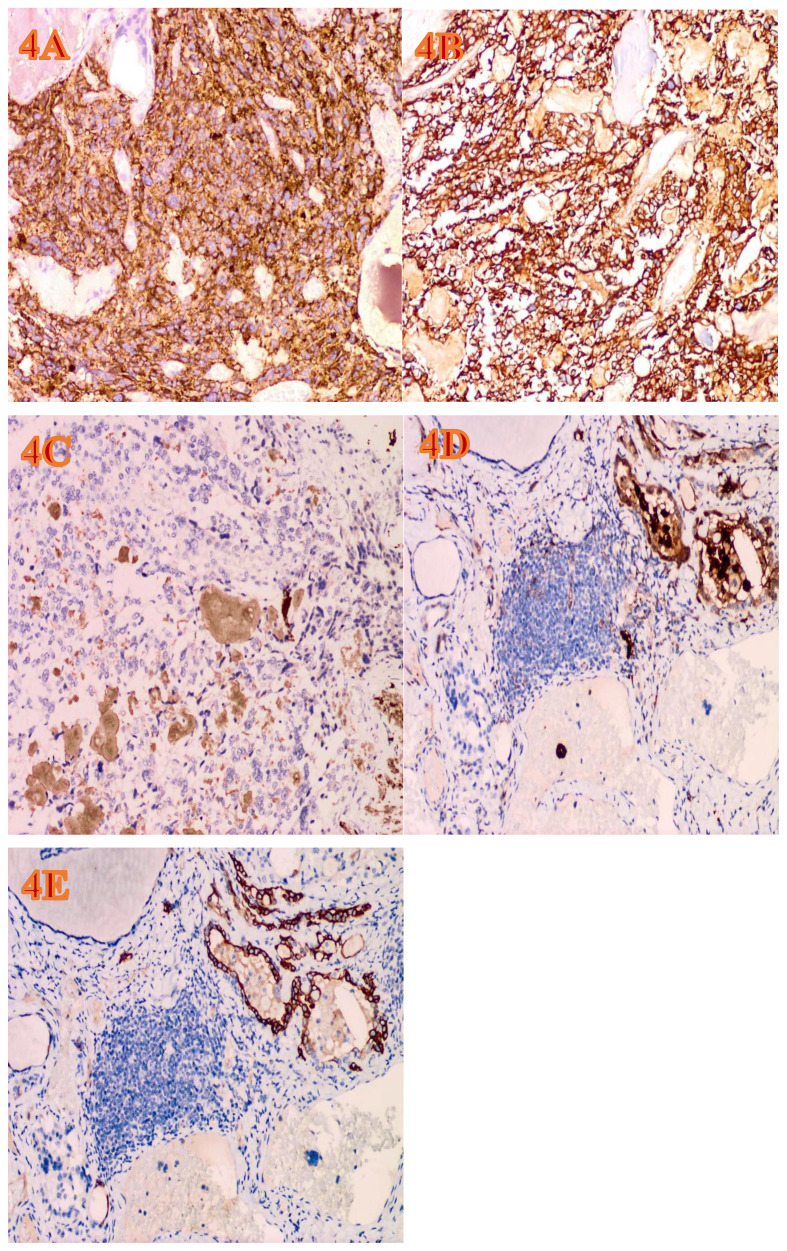
The immunohistochemical expression of the tumor (EnVision Method, H&E, ×200, magnification). **(A)** The area of MTC shows diffuse and strong positivity for synaptophysin. **(B)** The area of MTC shows diffuse and strong positivity for CEA. **(C)** Weakly positive CT focus in the MTC area. **(D)** Galectin-3 is positive in the PTC area. **(E)** CK19 is positive in the PTC area.

The gross appearance of the postoperative radical specimen shows: A gray-brown mass, measuring 4.5 cm x 3.3 cm x 2.5 cm, is found adjacent to the capsule of the right lobe of the thyroid gland(**Figure 2**). The cut surface is grayish-white and grayish-brown, solid, and of medium consistency. Calcification is seen in some areas. Two grayish-white nodules were seen adjacent to the capsule in the isthmus, with diameters of 0.4 cm and 0.5 cm respectively. The cut surfaces were grayish-white, solid and of medium consistency. A grayish-white nodule was seen adjacent to the capsule in the left lobe of the thyroid gland, with a diameter of 0.2 cm. The cut surface was grayish-white, solid and of medium consistency. Under H&E microscopy, the tumor in the right lobe of the thyroid gland shows unclear boundaries and no definite capsule. The thyroid capsule is invaded. The tumor is dominated by MTC with a small amount of classic PTC mixed in (**Figure 3A**). In the MTC area, tumor cells are arranged in sheets and nests, and trabeculae. A large amount of amyloid deposition is seen in the stroma. Multiple foci show calcification. Tumor cells have diverse shapes, including round, oval, and polygonal. Abnormal large cells and binucleated or multinucleated cells are observed. The nuclei are of varying sizes, with irregular nuclear membranes. The nuclear chromatin is granular (salt and pepper-like), and the nucleoli are prominent (**Figure 3B**). The proportion of PTC is less than 10%, located at the edge of the tumor lesion, interwoven with medullary carcinoma. It has typical PTC structure and nuclear features. The cells are closely arranged, the cytoplasm is ground-glass-like, the nuclei are round or oval, and nuclear overlap, nuclear grooves and intranuclear pseudoinclusions can be seen (**Figure 3C**). Immunohistochemistry (ELISON method, see **Figure 4**) showed: in the medullary carcinoma area: PCK (+), CEA (+), CT (focal cytoplasmic weak +), INSM1 (+), Syn (+), CgA (+), Galectin-3 (-), TG (-), CK19 (-), Ki-67 (+, 3%). In the PTC area: Galectin-3 (+), TG (+), CK19 (+), Syn (-), CT (-). The final diagnosis of the mass in the right lobe of the thyroid gland is MMPTC, with involvement of the capsule observed, but no vascular tumor thrombus or nerve invasion was found. Two nodules in the isthmus of the thyroid gland were both MTC, with microscopic diameters of 0.4 cm and 0.5 cm respectively, and neither involved the thyroid capsule. A focus of MTC (0.2 cm in diameter) and a focus of micro PTC (0.1 cm in diameter) were found in the left lobe of the thyroid gland, separated by normal thyroid tissue, and neither invaded the thyroid capsule. The lymph node dissection results showed that a total of 88 lymph nodes were dissected, among which 21 were found to have cancer metastasis. In the central lymph nodes, 8 were found to have cancer metastasis, and in the right cervical lateral lymph nodes, 13 were found to have cancer metastasis. Specifically, 5 cervical lateral lymph nodes were found to have MMPTC metastasis, and 5 central lymph nodes were found to have MMPTC metastasis; 4 cervical lateral lymph nodes were found to have simple MTC metastasis, and 3 central lymph nodes were found to have simple MTC metastasis; 2 cervical lateral lymph nodes were found to have simple PTC metastasis, and Simultaneous metastasis of both MTC and PTC was identified in 2 cervical lateral lymph nodes. The final pathological stage (AJCC 8th edition) was pT3aN1b. The patient’s serum CEA level dropped to normal after the operation, and the CT results were significantly better than before the operation. Postoperatively, the patient was treated with levothyroxine tablets to supplement thyroid hormones. Considering that the patient’s tumor was mainly composed of MTC, it is recommended that the patient undergo RET and BRAF gene somatic testing to evaluate the prognosis and provide a basis for targeted therapy. The patient indicated that they intended to go home for rest first and would undergo further testing later. Recently, somatic cell-based BRAF gene testing was performed on the papillary carcinoma region, and somatic cell-based RET gene testing was conducted on the medullary carcinoma region. The results demonstrated that mutations were present in the BRAF gene, whereas no mutations were detected in the RET gene. Currently, the patient is under continuous follow-up.

## Discussion

Reviewing the previous literature, it was found that the coexistence of MTC and PTC can be classified into four types (9): (Type I) True mixed MMPTC, where MTC and PTC are closely mixed. (Type II) Collision MTC/PTC, where the tumors meet at the same site and invade each other, presenting as a single mass in the thyroid gland, i.e., MTC and PTC coexist. (Type III) Tumors that are anatomically separate in the same lobe of the thyroid gland, i.e., separated by non-neoplastic thyroid tissue. (Type IV) Synchronous tumors occurring in different anatomical lobes or isthmus. The main lesion in this case is a combination of Type I and Type III, with multiple MTCs in the isthmus and mixed lymph node metastasis also found. These tumors are estimated to account for less than 1% of all thyroid tumors, but due to the lack of data, the true incidence rate remains unclear (11).

The origin, incidence, clinicopathological features and prognosis of MMPTC remain unclear. Currently, there are mainly three types of hypotheses regarding its pathogenesis (12). First, the “stem cell origin theory”: it is believed that during embryonic development, the same stem cell differentiates into follicular epithelial cells and parafollicular cells, which then develop into a mixed tumor of PTC and MTC. Second, the “collision theory”: it is believed that two independent tumors coincidentally collide and mix in the same lesion. Third, the “hostage theory”: it is believed that follicular cells are trapped by MTC cells and stimulated to proliferate by secreted influencing factors. Genetic alterations acquired by follicular cells during proliferation lead to their transformation into follicular-derived tumors.

The majority of MMPTC patients seek medical attention due to palpable neck masses or incidental discovery of thyroid nodules during physical examinations (13). A small number of patients may present with hoarseness, dysphagia, lymphadenopathy, or dyspnea. Generally, the nodules are larger than those in PTC or MTC, with a predominance in middle-aged and elderly patients, and a slightly higher incidence in females than males. The diameter of the cancerous lesions ranges from 0.4 to 11 cm (14). Most cases of MMPTC show elevated levels of CT and CEA in serological tests. Therefore, when blood tests indicate elevated CEA and CT, FNAC should be arranged more actively. In addition, it should be noted that when there is a significant increase in serum CEA and CT but the biopsy result is PTC, be vigilant for the possibility of MMPTC. The peculiarity of this case lies in the elevated serum CEA and CT levels, and the thyroid FNAC suggesting MTC. However, only simple PTC metastasis was found in the lymph node puncture tissue, which also indicates that we need to be vigilant about the occurrence of MMPTC and PTC/MTC. Reviewing the literature reveals that ultrasound and CT of patients with MMPTC often show abnormalities. Among them, ultrasound examination is the preferred auxiliary examination method for thyroid diseases. Literature (15) indicates that ultrasound examination has unique advantages in the diagnosis of thyroid masses. MMPTC, compared with simple MTC, shows more significant malignant features and is more easily detected by ultrasound at an early stage.

The diagnosis of MMPTC requires a close combination of clinical history, serological tests, ultrasound and CT imaging examinations. Pathological diagnosis serves as the gold standard, mainly relying on histological morphology combined with immunohistochemical examination, supplemented by genetic testing, to ultimately reach an accurate pathological diagnosis and tumor classification. Most MMPTCs can only be diagnosed through postoperative pathological examination, and it is extremely rare to be confirmed by preoperative FNAC (16). This may be related to the fact that the main component of most MMPTCs is MTC. FNAC may only obtain the MTC component and it is difficult to detect the PTC component. Lymph node puncture may make up for this deficiency. This case is exactly such a situation. Before radical surgery, it was suggested that both MTC and PTC might exist simultaneously, thus avoiding the occurrence of missed diagnosis. Mixed components can also be seen in metastatic foci within lymph nodes. The presence of MMPTC metastasis in 10 lymph nodes in this case also indirectly confirms the existence of MMPTC in the primary thyroid site. Under light microscopy, MTC and PTC components are independently distributed in one or both thyroid lobes in MTC/PTC. Immunohistochemistry is of particular significance in the diagnosis of two types of cancer, but some special circumstances should also be noted. In the PTC area, Tg, Galectin-3, and CK19 are positive, while CT is negative; in the MTC area, most patients have positive CT and CEA, and neuroendocrine cancer markers such as Syn, CgA, and CD56 are mostly positive, while TG is generally negative. It is worth noting that in some cases, due to the loss of the ability of tumor cells to produce CT, the phenomenon of CT negative and CEA positive occurs. This is basically consistent with the results of Wang Hongqun et al. (17) on the clinical pathological characteristics and prognosis analysis of 269 cases of MTC. The article summarizes the positive rates of various immune markers in MTC: TTF-1 (99.0%+), CT (85.4%+), Syn (98.7%+), CgA (81.9%+), CEA (88.8%+), and the Ki-67 positive rate was ≤10% in 95.1% of the patients. In this case, the immunohistochemistry of MTC shows that CT has a focal weak positive expression, which may be related to the above factors, but the patient’s serum CT level is significantly elevated, which is consistent with the disease status.It is worth noting that TTF-1 can be expressed in both MTC and PTC, but its positive intensity in MTC is lower than that in follicular tumors, so it cannot be used to distinguish MTC from PTC (18). With regard to molecular testing, it is recommended to perform BRAF and RAS gene mutation analyses on somatic cells from patients with papillary thyroid carcinoma (PTC) and medullary thyroid carcinoma (MTC), respectively, while RET genetic testing should be conducted on both somatic and germline DNA. Among them, high iodine intake is a high-risk factor for BRAF gene mutations in PTC patients; RET gene testing can be performed for MTC patients, and RET mutations are closely related to tumor invasiveness and poor prognosis (19). RAS mutations may be related to racial and environmental factors (20).

At present, no standardized treatment plan for MMPTC has been established in domestic and international guidelines, and mainly surgical-based treatment methods are adopted (21). Some researchers (22) suggest that the diagnosis and treatment strategies for MTC should be referred to for handling MMPTC. Preoperative assessment of serum CT levels and cervical lymph nodes, as well as selection of a reasonable surgical scope, are of great significance for MMPTC. At the same time, there is no systematic and precise individualized treatment plan for PTC/MTC at present. The current treatment for PTC/MTC mainly relies on surgery. Therefore, standardized surgical procedures may be the most effective treatment method at present (23). In previous literature, most patients underwent total thyroidectomy plus central and bilateral cervical lateral lymph node dissection, while a small number of cases underwent subtotal thyroidectomy plus cervical lymph node dissection.In this case, the preoperative thyroid examination of the patient revealed suspected MTC components, accompanied by metastatic PTC in the cervical lymph nodes. Therefore, total thyroidectomy plus central and bilateral cervical lateral lymph node dissection was chosen. This is consistent with the surgical approach selected in most previous related literature.

The Chinese Expert Consensus on Diagnosis and Treatment of MTC (2020 Edition) suggests (24): After surgery, an individualized treatment plan should be formulated based on the stratification of tumor recurrence risk and the risk of adverse reactions to thyroid-stimulating hormone suppression therapy. Comprehensive management should be carried out based on the assessment of TG levels and imaging examinations, etc. There is some controversy over whether MMPTC and PTC/MTC patients should receive iodine-131 treatment (25). MTC is a tumor derived from C cells and does not take up iodine itself. However, the effect of iodine treatment on the small amount of PTC component mixed in MTC remains unknown. There is literature (26) suggesting that when the PTC component is classified as medium to high risk according to the ATA risk stratification, radioactive iodine therapy should be administered even if it is ineffective for MTC. In this case, the PTC lesion was small, with the maximum diameter less than 1 cm. According to the ATA risk stratification criteria, it belongs to the low-risk group and thus does not meet the indications for radioactive iodine therapy. The treatment principle is similar to that of MTC. Some studies have suggested that if there are residual lesions that cannot be removed by the naked eye in PTC or MTC, external beam radiotherapy is also recommended for the patients (27, 28). In this case, the patient has undergone complete tumor resection and no residual lesions were found after the operation, so radiotherapy is not necessary.

Regarding the prognosis of MMPTC, the proportion of MTC and PTC components is an important consideration. Those with a predominance of MTC have a prognosis similar to that of MTC. A large-sample retrospective study by the US NCDB (29) showed that compared with PTC, MMPTC patients were older, and the 10-year survival rate of MMPTC was lower than that of PTC (76.04% vs 89.04%), but higher than that of MTC (70.29%). Among the clinical and pathological features analyzed by them, all-cause mortality was associated with the following factors: age, gender, race, stage, multifocality, extrathyroidal invasion, radiotherapy, and surgical approach.

## Conclusion

MMPTC and PTC/MTC are rare types of malignant thyroid tumors. Preoperative color Doppler ultrasound examination of the thyroid and cervical lymph nodes, as well as serum CT, CEA and TG tests, are of great guiding significance for the diagnosis of the disease and the determination of the surgical scope. Due to their atypical morphological features, preoperative FNAC is prone to missed diagnosis, and lymph node puncture biopsy can be supplemented at this time to reduce preoperative missed diagnosis. If the pathological sampling is insufficient or there is insufficient understanding of the disease, it may lead to missed diagnosis or misdiagnosis, and affect the staging judgment and prognosis evaluation of the disease. The treatment decisions of this type of disease are closely related to the proportion of MTC components, and its prognosis is between MTC and PTC. After surgery, the levels of serum CT and TG should be regularly monitored to detect early signs of tumor recurrence.

## Data Availability

The original contributions presented in the study are included in the article/supplementary material. Further inquiries can be directed to the corresponding author.
